# Intravenous ascorbic acid to prevent and treat cancer-associated sepsis?

**DOI:** 10.1186/1479-5876-9-25

**Published:** 2011-03-04

**Authors:** Thomas E Ichim, Boris Minev, Todd Braciak, Brandon Luna, Ron Hunninghake, Nina A Mikirova, James A Jackson, Michael J Gonzalez, Jorge R Miranda-Massari, Doru T Alexandrescu, Constantin A Dasanu, Vladimir Bogin, Janis Ancans, R Brian Stevens, Boris Markosian, James Koropatnick, Chien-Shing Chen, Neil H Riordan

**Affiliations:** 1 Department of Orthomolecular Studies, Riordan Clinic, 3100 N Hillside, Wichita, Kansas, 67210, USA; 2 Department of Regenerative Medicine, Medistem Inc, 9255 Towne Centre Drive, San Diego, California, 92121. USA; 3 Department of Medicine, Moores Cancer Center, University of California San Diego, 3855 Health Sciences Dr, San Diego, California, 92121, USA; 4 Department of Immunology, Torrey Pines Institute for Molecular Studies, 3550 General Atomics Court, La Jolla, California,92121, USA; 5 Department of Human Development, Nutrition Program, University of Puerto Rico, Medical Sciences Campus, San Juan, 00936-5067, PR; 6 Department of Pharmacy Practice, University of Puerto Rico, Medical Sciences Campus, School of Pharmacy, San Juan, 00936-5067, PR; 7 Department of Experimental Studies, Georgetown Dermatology, 3301 New Mexico Ave, Washington DC, 20018, USA; 8 Department of Hematology and Oncology, University of Connecticut, 115 North Eagleville Road, Hartford, Connecticut, 06269, USA; 9 Department of Surgery, University of Latvia, 19 Raina Blvd, Riga, LV 1586, Latvia; 10 Department of Surgery, Microbiology, and Pathology, University of Nebraska Medical Center, 42nd and Emile, Omaha, Nebraska, 86198, USA; 11 Department of Microbiology and Immunology, and Department of Oncology, Lawson Health Research Institute and The University of Western Ontario, 1151 Richmond Street, London, Ontario, N2G 3M5, Canada; 12 School of Medicine, Division of Hematology and Oncology, Loma Linda University,24851 Circle Dr, Loma Linda, California, 92354, USA

## Abstract

The history of ascorbic acid (AA) and cancer has been marked with controversy. Clinical studies evaluating AA in cancer outcome continue to the present day. However, the wealth of data suggesting that AA may be highly beneficial in addressing cancer-associated inflammation, particularly progression to systemic inflammatory response syndrome (SIRS) and multi organ failure (MOF), has been largely overlooked. Patients with advanced cancer are generally deficient in AA. Once these patients develop septic symptoms, a further decrease in ascorbic acid levels occurs. Given the known role of ascorbate in: a) maintaining endothelial and suppression of inflammatory markers; b) protection from sepsis in animal models; and c) direct antineoplastic effects, we propose the use of ascorbate as an adjuvant to existing modalities in the treatment and prevention of cancer-associated sepsis.

## Personal Perspective

Having worked in the area of cancer research for over a decade, the major focus of one of the authors' investigations has been to develop therapeutic solutions by using siRNA to directly inhibit growth of tumors [1], and to stimulate tumor immunity using antigen-specific vaccines [2-4] or unorthodox immune-modulatory approaches [5-9]. Not until the author's mother passed away from leukemia did he realize that, while many options have been developed in the treatment of cancers, relatively little can be performed at end-of-life. While life support technologies have significantly increased life span, the quality of life at end stages can be devastatingly poor. The author (whose training was in the basic research space) was surprised to realize that, for the majority of cancers, the patient is literally "waiting to die" while on various supportive measures.

This led to the realization that there is a major need for supportive steps that: increase the quality of life, "do no harm", and hold out the possibility (however slim) of restoring some measure of lost life functions back to patients. One intervention that caught the attention of the author while at his mother's bedside was the practice of intravenous ascorbic acid (IV AA) administration [10,11]. That specific intervention was supported by a report in the literature that intravenous administration of AA (10g twice and 4 g daily orally for one week)significantly increased the quality of life in end stage patients [12]. Could such an easy-to-implement therapy actually be of benefit to patients facing the same challenges of the deceased mother of the author?

When the author discussed this option with others, it became evident that the value of i.v. AA in cancer treatment is controversial. In the 1970 s work by Cameron and Pauling demonstrated an approximate 4-fold survival increase in terminal cancer patients administered AA by i.v. and oral routes, compared to historical controls [13,14], a finding that was also observed in the results of a trial published by Murata *et al. *[15]. Subsequent trials that did not use historical controls but had a double-blind placebo-controlled design failed to find benefit [16,17]. The controversy has continued with recent reports that oral AA administration, which was used in the trials that failed to demonstrate benefit, fails to increase plasma concentrations to a level estimated to be sufficient to induce tumor cytotoxicity [18-24]. Currently, i.v. AA is used extensively by "alternative medicine" practitioners in the USA (11,233 patients treated in 2006 and 8876 patients in 2008) [25], although the basis for this practice has not been adopted into mainstream medicine. It is our belief that, in the practice of medicine, opinion should not hold greater weight than evidence - either a treatment has beneficial effects or it does not, and it is that consideration that must drive practice. We therefore sought, not to address the controversial area of whether AA shrinks tumors (which is currently being addressed in ongoing FDA approved trials [26-31]), but instead in an area that we feel has been highly under-explored: that is, suppression of inflammation in the cancer patient. In the context of cancer, inflammation may be seen as a continuum of possible degrees of severity ranging from low level, chronic inflammatory response to acute, highly severe inflammation. At the chronic end, low grade inflammation causes a variety of pathologies to the patient, perhaps most profound of which is cachexia [32-35], but also other effects such as poor post-surgical outcomes [36,37]. At the other end of the spectrum is the acute inflammation observed in the systemic inflammatory response syndrome (SIRS), a major cause of death of cancer patients and especially patients with hematological malignancies [38-40]. While we focus in this paper on SIRS and cancer, some of the concepts discussed are also applicable to chronic inflammatory conditions.

## What is SIRS?

According to the accepted definition, Systemic Inflammatory Response Syndrome (SIRS) is a term characterizing an inflammatory syndrome caused by infectious or traumatic causes in which patients exhibit at least 2 of the following criteria: 1) Body temperature less than 36°C or greater than 38°C; 2) Heart rate greater than 90 beats per minute; 3)Tachypnea, with greater than 20 breaths per minute; or, an arterial partial pressure of carbon dioxide less than 4.3 kPa (32 mmHg: 4) White blood cell count less than 4000 cells/mm^3 ^(4 × 109 cells/L) or greater than 12,000 cells/mm^3 ^(12 × 109 cells/L); or the presence of greater than 10% immature neutrophils (band forms) [41]. SIRS is different than sepsis in that in sepsis an active infection is found [42]. These patients may progress to acute kidney or lung failure, shock, and multiple organ dysfunction syndrome. The term septic shock refers to conditions in which the patient has a systolic blood pressure of less than 90 mmHg despite sufficient fluid resuscitation and administration of vasopressors/inotropes.

Predominant events in the progression to SIRS and subsequently to MOF include: a) systemic activation of inflammatory responses [43]; b) endothelial activation and initiation of the clotting cascade, associated with consumption of anticoagulants and fibrinolytic factors [44]; c) complement activation [45]; and d) organ failure and death. These pathological events appear to be related to each other, for example, it is known that complement activation stimulates the pro-coagulant state [46]. In the cancer patient SIRS may be initiated by several factors. Numerous patients receive immune suppressive chemo/radiotherapies that promote opportunistic infections [47,48]. Additionally, given that approximately 40-70% of patients are cachectic, the low grade inflammation causing the cachexia could augment effects of additional bacterial/injury-induced inflammatory cascades [49]. Finally, tumors themselves, and through interaction with host factors, have been demonstrated to generate systemically-acting inflammatory mediators such as IL-1, IL-6, and TNF-alpha that may predispose to SIRS [50,51].

Current SIRS treatments SIRS are primarily supportive. To date, the only drug to have elicited an effect on SIRS in Phase III double-blind, placebo-controlled trials has been Xigris (activated protein C (APC)) [52], which exerts its effects by activating endothelial cell-protecting mechanisms mediating protection against apoptosis, stimulation of barrier function through the angiopoietin/Tie-2 axis, and by reducing local clotting [53-55]. The basis of approval for Xigris has been questioned by some [56] and, additionally, it is often counter-indicated in oncology-associated sepsis (especially leukemias where bleeding is an issue of great concern). In fact, in the Phase III trials of Xigris, hematopoietic transplant patients were excluded [57]. Thus there is a great need for progress in the area of SIRS treatment and adjuvant approaches for agents such as Xigris.

## Endothelial Dysfunction of SIRS

One of the main causes of death related to SIRS is dysfunction of the microcirculatory system, which in the most advanced stages is manifested as disseminated intravascular coagulation (DIC) [44]. Inflammatory mediators associated with SIRS, whether endotoxin or injury-related signals such as TLR agonists or HMGB-1, are all capable of activating endothelium systemically [58,59]. Under physiological conditions, the endothelial response to such mediators is local and provides a useful mechanism for sequestering an infection and allowing immune attack. In SIRS, the fact that the response is systemic causes disastrous consequences includingorgan failure. The characteristics of this endothelial response include: a) upregulation of tissue factor (TF) [60,61] and suppression of endothelial inhibitors of coagulation such as protein C and the antithrombin system causing a pro-coagulant state [62]; b) increased expression of adhesion molecules which elicit, in turn, neutrophil extravasation [63]; c) decreased fibrinolytic capacity [64-66]; and d) increased vascular permeability/non-responsiveness to vaso-dilators and vasoconstrictors [67,68]. Excellent detailed reviews of molecular signals associated with SIRS-induced endothelial dysfunction have been published[69-77] and one of the key factors implicated has been NF-kB [78]. Nuclear translocation of NF-kB is associated with endothelial upregulation of pro-thrombotic molecules and suppressed fibrinolysis [79-81]. In an elegant study, Song *et al*. inhibited NF-kB selectively in the endothelium by creation of transgenic mice transgenic expressing exogenous i-kappa B (the NF-kB inhibitor) specifically in the vasculature. In contrast to wild-type animals, the endothelial cells of these transgenic mice experienced substantially reduced expression of tissue factor while retaining expression of endothelial protein C receptor and thrombomodulin subsequent to endotoxin challenge. Furthermore, expression of NF-B was associated with generation of TNF-alpha as a result of TACE activity [82].

It is interesting that the beneficial effects of Xigris in SIRS appear to be associated with its ability to prevent the endothelial dysfunction [83] associated with suppression of proinflammatory chemokines [84], prevention of endothelial cell apoptosis [85], and increased endothelial fibrinolytic activity [86,87]. Some of the protective activities of Xigris have been ascribed to its ability to suppress NF-kB activation in endothelial cells [88,89].

## Ascorbic Acid Effects on Endothelium

Several clinical studies have supported the possibility that AA mediates a beneficial effect on endothelial cells, especially in the context of chronic stress. Heitzer *et al. *[90] examined acetylcholine-evoked endothelium-dependent vaso-responsiveness in 10 chronic smokers and 10 healthy volunteers. While responsiveness was suppressed in smokers, administration of intra-arterial ascorbate was capable of augmenting reactivity: an augmentation evident only in the smokers. Endothelial stress induced in 17 healthy volunteers by administration of L-methionine led to decreased responsiveness to hyperemic flow and increased homocysteine levels. Oral AA (1 g/day) restored endothelial responsiveness [91]. Restoration of endothelial responsiveness by AA has also been reported in patients with insulin-dependent [92] and independent diabetes [93], as well as chronic hypertension [94]. In these studies AA was administered intraarterially or intravenously, and the authors proposed the mechanism of action to be increased nitric oxide (NO) as a result of AA protecting it from degradation by reactive oxygen species (ROS).

A closer look at the literature suggests that there are several general mechanisms by which AA may exert endothelial protective properties. The importance of basal production of NO in endothelial function comes from its role as a vasodilator, and an inhibitor of platelet aggregation [95,96]. High concentrations of NO are pathological in SIRS due to induction of vascular leakage [97]. However, lack of NO is also pathological because it causes loss of microvascular circulation and endothelial responsiveness [98,99]. Although there are exceptions, the general concept is that inducible nitric oxide synthase (iNOS) and neuronal nitric oxide synthase (nNOS) are associated with sepsis-induced pathologies, whereas eNOS is associated with protective benefits [100]. It is important to note that, while iNOS expression occurs in almost all major cells of the body in the context of inflammation, eNOS is constitutively expressed by the endothelium. AA administration decreases iNOS in the context of inflammation [101,102], but appears to increase eNOS [103]. Thus, AA appears to increase local NO concentrations through: a) prevention of ROS-mediated NO inactivation [104,105]; b) increased activity of endothelial-specific nitric oxide synthase (eNOS) [106], possibly mediated by augmenting bioavailability of tetrahydrobiopterin [107-112], a co-factor of eNOS [113]; and c) induction of NO release from plasma-bound S-nitrosothiols [103].

In addition to deregulation of NO, numerous other endothelial changes occur during SIRS, including endothelial cell apoptosis, upregulation of adhesion molecules, and the procoagulant state [114]. AA has been reported to be active in modulating each of these factors. Rossig *et al*. reported that *in vitro *administration of AA led to reduction of TNF-alpha induced endothelial cell apoptosis [109]. The effect was mediated in part through suppression of the mitochondria-initiated apoptotic pathway as evidenced by reduced caspase-9 activation and cytochrome c release. To extend their study into the clinical realm, the investigators prospectively randomized 34 patients with NYHA class III and IV heart failure to receive AA or placebo treatment. AA treatment (2.5 g administered intravenously and 3 days of 4 g per day oral AA) Resulted in reduction in circulating apoptotic endothelial cells in the treated but not placebo control group [115]. Various mechanisms for inhibition of endothelial cell apoptosis by AA have been proposed including upregulation of the anti-apoptotic protein bcl-2 [116] and the Rb protein, suppression of p53 [117], and increasing numbers of newly formed endothelial progenitor cells [118].

AA has been demonstrated to reduce endothelial cell expression of the adhesion molecule ICAM-1 in response to TNF-alpha *in vitro *in human umbilical vein endothelial (HUVEC) cells (HUVEC) [119]. By reducing adhesion molecule expression, AA suppresses systemic neutrophil extravasation during sepsis, especially in the lung [120]. Other endothelial effects of AA include suppression of tissue factor upregulation in response to inflammatory stimuli [121], and effect expected to prevent the hypercoaguable state. Furthermore, ascorbate supplementation has been directly implicated in suppressing endothelial permeability in the face of inflammatory stimuli [122-124], which would hypothetically reduce vascular leakage. Given the importance of NF-kappa B signaling in coordinating endothelial inflammatory changes [79-81], it is important to note that AA at pharmacologically attainable concentrations has been demonstrated to specifically inhibit this transcription factor on endothelial cells [125]. Mechanistically, several pathways of inhibition have been identified including reduction of i-kappa B phosphorylation and subsequent degradation [126], and suppression of activation of the upstream p38 MAPK pathway [127]. *In vivo *data in support of eventual use in humanshas been reported showing that administration of 1 g per day AA in hypercholesterolemic pigs results in suppression of endothelial NF-kappa B activity, as well as increased eNOS, NO, and endothelial function [128]. In another porcine study, renal stenosis was combined with a high cholesterol diet to mimic renovascular disease. AA administered i.v. resulted in suppression of NF-kappa B activation in the endothelium, an effect associated with improved vascular function [129].

An important factor in reports of clinical studies of AA is the difference in effects seen when different routes of administration are employed. Supplementation with oral AA appears to have rather minor effects, perhaps due to the rate-limiting uptake of transporters found in the gut. Indeed, maximal absorption of AA appears to be achieved with a single 200 mg dose [130]. Higher doses produce gut discomfort and diarrhea because of effects of ascorbate accumulation in the intestinal lumen [131]. This is why some studies use parenteral administration. An example of the superior biological activity of parenteral versus oral was seen in a study administering AA to sedentary men. Parenteral but not oral administration was capable of augmenting endothelial responsiveness as assessed by a flow-mediated dilation assay [132].

## Cancer Patients are Deficient in Ascorbic Acid

The general activity of AA as an anti-oxidant implies that conditions associated with chronic inflammation and oxidative stress would lead to its depletion. As reviewed by McGregor and Biesalski [133], numerous inflammatory conditions including gastritis [134], diabetes [134,135], pancreatitis [136], pneumonia [137], osteoporosis [138], rheumatoid arthritis [139], are all associated with marked reduction in plasma AA levels as compared to healthy controls. Within the context of this discussion, profound reduction of AA is observed in cancer patients [140-146], SIRS patients [147], and ICU patients [134].

Some studies have demonstrated correlation between plasma AA and survival. Mayland *et al. *[141] measured plasma AA in 50 end-stage cancer patients in a hospice setting. A correlation between deficiency in AA, decreased survival, and higher expression of the inflammatory marker CRP was noted. More recently, a correlation between tumor aggressiveness and low AA content has been made [148]. Kuiper *et al*. found that the proangiogenic transcription factor HIF-1 alpha is negatively correlated with tumor AA content. Correlations where also made between low AA content, high VEGF, and levels of the anti-apoptotic protein bcl-2.

Cancer patients are known to exhibit a general state of chronic inflammation which, as stated above, is related to the tumor itself and the interaction of host factors with the tumor. Elevation in the level o f classical inflammatory markers such as fibrinogen [149-155], CRP [156-160], erythrocyte sedimentation rate [161], ferritin [162-165], neopterin [166-168], homocysteine [169,170], IL-6 [161,171], and free radical stress [172-175] have been well-documented in cancer patients, with numerous studies demonstrating that elevation is associated with poor survival.

The possibility that inflammation itself reduces plasma AA was shown by Fain *et al. *[176], who examined 184 hospitalized patients and observed that 47.3% suffered from hypovitaminosis C as defined as either depletion (*i.e*., serum AA levels < 5 mg/l) or deficiency (*i.e*., serum AA levels < 2 mg/l). Interestingly, patients with an activated acute phase response, as defined by erythrocyte sedimentation rate above 20 mm and an increase in acute phase reactants (CRP >10 mg/l and/or fibrinogen > 4 g/l) had lower serum AA levels. Also associated with decreased serum AA levels was reduction in hemoglobin and albumin. A Japanese population study of 778 men and 1404 women, aged 40-69 years, demonstrated a negative correlation between plasma AA content and CRP [177]. In an interventional study, Block *et al*. examined 396 healthy nonsmokers randomized to receive either 1000 mg/day vitamin C, 800 IU/day vitamin E, or placebo, for 2 months. A statistically significant decrease in plasma CRP levels was found only in the group receiving AA [178].

While a study by Mayland *et al*. demonstrated that, in 50 patients with advanced malignancies of various types, a correlation between high CRP levels and AA deficiency existed [179], to our knowledge no interventional studies in cancer patients have been performed to assess the capacity of AA administered i.v. to inhibit chronic inflammation. In the absence of such studies, we looked at reports of AA inhibition ofs inflammatory markers in the context of other diseases to determine whether a rationale may exist for its use in cancer. Several such supporting studies exist. Administration of IV AA has been shown to decrease CRP levels in smokers [180]. Oral AA supplementation decreased CRP levels in a trial of 44 patients suffering from atrial fibrillation after cardioversion [181]. In a study of 12 healthy volunteers, it was shown that i.v. AA inhibited endothelin-induced IL-6 production [182]. In a study of 1463 coronary artery disease patients, a negative correlation between neopterin (a catabolic product of GTP indicative of immune activation) and AA concentration was noted [183]. Given that there are, at present, numerous trials being conducted using i.v. AA in the treatment of cancer [26-31], it is highly unfortunate that none of them are assessing inflammatory markers or other potential mechanisms of action. This may, to some degree, be detrimental to future study of AA in cancer treatment: if poor tumor regression data is generated, replication of these trials with inclusion of sensitive inflammatory marker endpoints may never occur.

## SIRS patients are deficient in AA

The progression of SIRS into MOF is perhaps one of the most inflammation-driven disease pathologies. If the overall hypothesis that AA is consumed by inflammation is correct, these patients should be highly deficient. This appears to be the case: several studies have demonstrated severe deficiency in AA in patients with sepsis and septic shock compared to healthy volunteers. Doise *et al*. examined 37 patients with septic shock, 19 patients with severe sepsis, and 6 healthy volunteers over the period of 10 days. A significant deficiency of AA was observed compared to controls, and blood AA levels continued to decline while the patients were in the ICU. No difference between the deficiency in septic shock and severe sepsis was noted [184]. The association ofAA deficiency with poor outcomes was further strengthened in a study of 16 ICU patients in which a statistically significant decrease in AA was found in patients progressing to MOF [185]. Indeed, septic patients have been demonstrated to exhibit a much higher rate of ascorbate consumption compared to healthy volunteers, based on studies in which predefined doses of AA were administered and *in vivo *degradation and disappearance was assessed [186].

Animal models suggest a critical role for AA in protecting from/inhibiting the septic process. In an elegant study, mice deficient for ascorbic acid synthesis (*i.e*., deficient in L-gulono-gamma-lactone oxidase) were depleted of exogenous ascorbate by feeding on an ascorbate-free diet and challenge with the pathogen *Klebsiella pneumonia*. Mortality was 3-fold higher in ascorbate-deficient animals compared to controls, which received a standard ascorbate-containing diet [187]. Given that cancer patients are generally deficient in AA, these findings may suggest the importance of maintaining at least normal AA levels to prevent from onset of SIRS [140-146]. Supplementation with AA has been demonstrated to protect against sepsis-associated death. Using a "feces injection into the peritoneum" model of sepsis, i.v. injection of 10 mg/kg AA resulted in 50% survival, in contrast to a 19% survival in animals receiving saline [98]. Supplementation with AA improved outcome in sepsis-associated hypoglycemia [188], microcirculatory abnormalities [189], and blunted endothelial responsiveness [101,102,190] in animal models.

From a clinical perspective, Crimi *et al*. reported a prospective randomized study in which vitamins C (500 mg/d) and E (400 IU/d) where administered via enteral tube to a group of 105 critically ill patients, whereas a control group of 111 patients received a isocaloric formula without supplementation with these vitamins. At patient follow-up, reduced TBARS and isoprostanes (markers of oxidative stress) were observed in the treated group. In addition, improved survival at 28 days of treatment was reported: 54.3% in the antioxidant group and 32.5% in the regular-feeding group (*p *< 0.05) [191]. Nathens *et al*. performed a larger study of 595 critically ill surgical patients where the majority suffered from trauma. AA and vitamin E where administered i.v. 3 times per day (1000 mg per injection and 1000 IU enterally, respectively). Reductions in the time of hospital stay, pulmonary mortality, and need for mechanical ventilation was observed in the treated group. Furthermore, MOF incidence was reduced in the anti-oxidant supplemented group [192]. In a study of the effect of AA alone in treatment of burn patients with > 30% of their total body surface area affected, patients were given AA i.v. (66 mg/kg/hr for 24 hours, n = 19) or received only standard care (controls, n = 18). AA treatment resulted in statistically significant reductions in 24 hr total fluid infusion volume, fluid retention (indicative of vascular leakage), and MDA. Perhaps most striking was the decrease in the need for mechanical ventilation: the treated group required an average of 12.1 ± 8.8 days, while the control group required 21.3 ± 15.6 days [193].

Thus it appears that cancer patients generally have a deficiency in AA which may predispose to SIRS and subsequent MOF, and patients with other diseases exhibit symptom severity inversely associated with AA levels. Patients who do develop SIRS and MOF have even greater depletion of AA and, as a result, various changes in the endothelium occur which exacerbate progression to mortality. Thus, there is some rationale for use of AA in cancer patients to prevent/treat SIRS. There is an additional possible benefit in that AA may actually inhibit cancer initiation and growth. Without providing an exhaustive review of this controversial subject, we will touch upon some work that has been performed in this area.

## AA Effects in Cancer

The state of AA deficiency in cancer patients, whether or not as a result of inflammation, suggests that supplementation may yield benefit in quality of life. Indeed, this was one of the main findings that stimulated us to write this review [12]. Improvements in quality of life were also noted in the early studies of Murata *et al. *[15] and Cameron [11]. But, in addition to this endpoint, there appears to be a growing number of studies suggesting direct anti-cancer effects via generation of free radicals locally at tumor sites [21]. *In vitro *studies on a variety of cancer cells including neuroblastoma [194], bladder cancer [195], pancreatic cancer [196], mesothelioma [197], and hepatoma [198], have demonstrated cytotoxic effects at pharmacologically-achievable concentrations. Enhancement of cytotoxicity of docetaxel, epirubicin, irinotecan, and 5-FU to a battery of tumor cell lines by AA was demonstrated *in vitro *[199]. *In vivo *studies have also supported the potential anticancer effects of AA. For example, Pollard *et al*. used the rat PAIII androgen-independent syngeneic prostate cancer cell line to induce tumors in Lobund-Wistar rats. Daily intraperitoneal administration of AA for 30 days (with evaluation at day 40) revealed significant inhibition of tumor growth and reduction in pulmonary and lymphatic metastasis [200]. Levine's group reported successful in vivo inhibition of human xenografted glioma, overian, and neuroblastoma cells in immune-deficient animals by administration of AA. Interestingly, control fibroblasts were not affected [23]. Clinical reports of remission induced by i.v. AA have been published [201]. However, as mentioned above, formal trials are still ongoing. Table 1 summarizes previous trials.

**Table 1:** Ascorbic Acid Cancer Trials

Condition	Number of Patients	Dose/Route	Finding	Ref
Mixture of solid tumors at different stages	49	Intravenous for 10 days 10 g and subsequently daily oral 10 g/day	17 pts no response, 10 pts minimal response, 11 pts growth retardation, 2 pts cytostasis, 5 pts tumor regression, 4 pts tumor hemorrhage/necrosis	11

Terminal cancer patients	39	Intravenous 10 g vitamin C twice with a 3-day interval and an oral intake of 4 g vitamin C daily for a week	Health score improved from 36+/-18 to 55+/-16 (p = 0.001). Significantly higher scores for physical, role, emotional, and cognitive function (p < 0.05). In symptom scale, the patients reported significantly lower scores for fatigue, nausea/vomiting, pain, and appetite loss (p < 0.005).	12

Terminal cancer patients	100 cancer pts treated as compared to 1000 controls. 50 of the treated pts were in the publication described in ref 11.	Intravenous for 10 days 10 g and subsequently daily oral 10 g/day	Mean survival time > 4.2 times as great for the ascorbate subjects (more than 210 days) as for the controls (50 days). Survival-time curves indicate that deaths occur for about 90% of the ascorbate-treated patients at one-third the rate for the controls and that the other 10% have a much greater survival time, averaging more than 20 times that for the controls.	13

Terminal cancer patients	99 in one hospital and 31 in another hospital	30g/day intravenously	Hospital #1: Survival of 43 days for 44 low-ascorbate patients and 246 days for 55 high-ascorbate patients. Hospital #2: 48 days for 19 control patients and 115 days for 6 high-ascorbate patients.	15

Terminal cancer patients	60 AA, 63 placebo controlled	10 g/day oral	The two groups showed no appreciable difference in changes in symptoms, performance status, appetite or weight. The median survival for all patients was about seven weeks, and the survival curves essentially overlapped.	16

Advanced colorectal cancer	50 AA, 50 control	10 g/day oral	AA treatment had advantage over placebo with regard to either the interval between the beginning of treatment and disease progression or patient survival. Among patients with measurable disease, none had objective improvement.	17

Renal metastatic, B cell lymphoma, Bladder cancer	3 Cases	50-100 g intravenously, various regimens	Tumor regression and unexpectedly long survival.	201

In addition to direct cytotoxicity of AA on tumor cells, inhibition of angiogenesis may be another mechanism of action. It has been reported that AA inhibits HUVEC proliferation *in vitro *[202] and suppresses neovascularization in the chorionic allontoic membrane assay [203]. We recently reported that *in vivo *administration of AA suppresses vascular cord formation in mouse models [204]. Supporting this, Yeom *et al*. demonstrated that parenteral administration of AA in the S-180 sarcoma cell model leads to reduced tumor growth, which was associated with suppression of angiogenesis and reduced expression of the pro-angiogenic factors bFGF, VEGF, and MMP-2 [205]. Recent studies suggest that AA suppresses activation of the hypoxia-inducible factor (HIF)-1, which is a critical transcription factor that stimulates tumor angiogenesis [206-208]. The clinical relevance of this has been demonstrated in a study showing that endometrial cancer patients with reduced tumor ascorbate levels have higher levels of active HIF-1 and a more aggressive phenotype [148].

Thus the possibility exists that administration of AA for treatment of tumor inflammation-mediated pathologies may also cause an antitumor effect. Whether this effect is mediated by direct tumor cytotoxicity or inhibition of angiogenesis remains to be determined. Unfortunately, none of the ongoing trials of AA in cancer patients seek to address this issue [26-31].

## Areas needing study: AA and Immunity

Despite numerous claims in the popular media (and even on labels on over-the-counter vitamin packaging), AA stimulation of immune function to reduce tumor initiation and growth is not clear-cut. This is partly because ROS are involved in numerous signaling events in immune cells [209]. For example, it is known that T cell receptor signaling induces an intracellular flux of ROS which is necessary for T cell activation [210]. There are also numerous studies demonstrating that ascorbic acid, under certain conditions, can actually inhibit immunity. For example, high dose ascorbate inhibits T cell and B cell proliferative responses as well as IL-2 secretion *in vitro *[211,212], and NK cytotoxic activity [213]. In addition, AA has been demonstrated to inhibit T cell activation of dendritic cells by encouraging them to remain in an immature state, in part through inhibition of NF-kappa B [214].

It is possible, although not formally tested, that the immune stimulatory effects of AA are actually observed in the context of background immune suppression or in situations of AA deficiency, both of which are well-known in the cancer and SIRS patient. Cleavage of the T cell receptor (TCR) zeta chain is a common occurrence in cancer [215-219] and SIRS patients [220,221]. The zeta chain is an important functional factor in T cell and NK cell activation, and is the most highly expressed of the immunoreceptor tyrosine-based activation motifs (ITAMs) on T and NK cells [222]. At the cellular level, cleavage of the zeta chain is associated with loss of T/NK cell function and spontaneous apoptosis [223-225] and, in the clinic, it is associated with poor prognosis [226-231].

Since loss of the TCR zeta chain is found in other inflammatory conditions ranging from hemodialysis [232,233], to autoimmunity [234-237], to heart disease [238], the possibility that inflammatory mediators such as ROS cause TCR zeta downregulation has been suggested. Circumstantial evidence comes from studies correlating presence of inflammatory cells such as tumor-associated macrophages with suppression of zeta chain expression [239]. Myeloid suppressor cells (which are known to produce high concentrations of ROS [240-242]) have also been demonstrated to induce reduction of TCR zeta chain in cancer [243], and after trauma [244]. Administration of anti-oxidants has been shown to reverse TCR zeta chain cleavage in tissue culture [245,246]. Therefore, from the T cell side of immunity, an argument could be made that intravenous ascorbic acid may upregulate immunity by blocking zeta chain downregulation in the context of cancer and acute inflammation.

While it is known that AA functions as an antioxidant in numerous biological conditions, as well as reduces inflammatory markers, the possibility that AA actually increases immune function in cancer patients has never been formally tested. This is an area that in our opinion cries out for further studies.

## Conclusion

AA administered intravenously has a long and controversial history in relation to reducing tumors in patients. This has impeded research into other potential benefits of this therapy in cancer patients such as reduction of inflammation, improvement of quality of life, and reduction ofSIRS initiation and progression to MOF. While ongoing clinical trials of i.v. AA for cancer may or may not meet the bar to grant this modality a place amongst the recognized chemotherapeutic agents, it is critical that we collect as much biological data as possible, given the possibility of this agent to be a wonderful adjuvant therapy.

## Competing interests

The authors declare that they have no competing interests.

## Authors' contributions

TEI, BM, TB, BL, RH, NAM, JAJ, MJG, JRMM, DTA, CD, VB, JA, RBS, BM, JK, CSC, NHR all contributed to the development of the concept, literature review, discussions, and writing of the manuscript. All authors have read the manuscript and agree to its submission.
